# Role of IFIT1 and IFIT3 in systemic lupus erythematosus: modeling a diagnosis and exploring immune regulation

**DOI:** 10.1007/s10238-026-02265-6

**Published:** 2026-07-24

**Authors:** Luofei Huang, Jian Shi

**Affiliations:** 1 Department of Internal Medicine, Liuzhou Municipal Liutie Central Hospital, Liuzhou, 545000 Guangxi China; 2 Department of Internal Medicine, The People’s Hospital of Laibin, Laibin, 546199 Guangxi China

**Keywords:** Systemic lupus erythematosus, Single-cell sequencing, Mendelian randomization, Expression quantitative trait loci, Weighted gene co-expression network analysis

## Abstract

Systemic lupus erythematosus (SLE) is a complex autoimmune disorder characterized by multi-organ involvement and a protracted clinical course. Current diagnostic strategies, which rely heavily on clinical symptoms and serology, are often insufficient for early detection. Therefore, highly accurate diagnostic biomarkers are urgently needed to facilitate early intervention and optimize personalized treatment strategies. D atasets GSE61635 and GSE135779 were integrated to identify differentially expressed genes. Weighted gene co-expression network analysis (WGCNA) was performed to isolate the module with the strongest clinical relevance. Mendelian randomization and single‑cell RNA‑seq were used to identify key disease‑relevant genes. A diagnostic model was then constructed, and gene set variation analysis (GSVA), along with gene set enrichment analysis (GSEA), was conducted to elucidate the underlying molecular pathways. *IFIT1* and *IFIT3* were identified as 2 core genes highly expressed in monocytes and T cells of SLE patients. Functional enrichment analysis revealed that these genes were enriched in immune-related pathways, metabolic pathways related to inflammation and genomic stability. The diagnostic model showed good accuracy, with an area under the curve (AUC) of 0.974 on the training set and 0.912 on the validation set. *IFIT1* and *IFIT3* represent promising biomarkers for diagnosing SLE and appear to mediate key immune and metabolic disturbances. Furthermore, the developed model serves as an accurate and reliable instrument for early diagnosis and personalized therapy. Large-scale clinical studies are warranted to further validate these findings and evaluate their clinical application.

**Supplementary Information:**

The online version contains supplementary material available at https://doi.org/10.1007/s10238-026-02265-6.

## Introduction

Systemic lupus erythematosus (SLE) is a chronic inflammatory autoimmune disorder that causes inflammation and tissue damage throughout the body, affecting many systems, including the skin, kidneys, joints and other organs [1–3]. Despite extensive research into the pathogenesis of this disease, its molecular mechanisms are still not fully understood. At present, the diagnosis of SLE is based mainly on clinical manifestations and laboratory markers, although these are often nonspecific and fail to capture the disease’s complexity [4]. The Type I interferon signature (IFN-score) is often used in SLE research; however, its clinical applications have encountered multiple limitations. For instance, interferon scoring assays across multiple genes incur high testing costs and are poorly standardized. Moreover, reproducibility across laboratories and patients remains controversial. In addition to these practical obstacles, a single IFN score exhibits limited ability to fully capture the complex immune heterogeneity in SLE. Thus, identifying a small number of core, stably expressed, easily measured ISGs as surrogate biomarkers has enormous potential for clinical translation. There remains a critical, unfulfilled demand for precise, accessible biomarkers capable of facilitating early diagnosis, tracking disease progression, and guiding targeted therapeutic decisions [5].

In recent years, advancements in high-throughput genomic technologies such as transcriptomics and single-cell RNA sequencing (scRNA-seq) have provided new insights into the molecular landscape of autoimmune diseases, such as SLE [6]. These methods enable whole-body gene expression profiling of bulk tissue and single cells, which uncover subtle differences in immune cell activity that could be key to disease onset and development [7]. A growing body of evidence suggests that gene co-expression networks and Mendelian randomization (MR) analyses are powerful approaches for discovery and for identifying potential disease genes and their causal relationships with clinical outcomes [8–11].

This study integrated multiple transcriptomic datasets from public repositories, including Gene Expression Omnibus (GEO)(GSE61635 and GSE135779), to explore gene expression profiles in SLE. Utilizing differential expression analysis, weighted gene co-expression network analysis (WGCNA), Mendelian randomization [12], and scRNA-seq [13]. we aimed to identify key diagnostic biomarkers, construct a predictive diagnostic model, and evaluate their clinical prognostic value. This integrative framework sought to elucidate the molecular mechanisms driving SLE, uncover potential therapeutic targets, improve diagnostic accuracy, and deepen our understanding of its complex immune pathogenesis.

## Research methodology and data sources

### Research design

For this study, we obtain the gene expression data set GSE61635 from GEO database (Home-GEO-NCBI, NIH) and do background correction for R [14]. Generally, we obtained the expression level of a gene by averaging the value of multiple probes from the same gene. DEGs were detected by “Limma” package [15].Next, we constructed a WGCNA based on the “WGCNA” package and made a scale-free topology network, converting the data matrix into adjacency matrix. By calculating the MEs of modules, merging similar modules by the hierarchical cluster tree of ME values, and we obtained a dendrogram for module relationship. The gene significance (GS) and the module significance (MS) were computed to calculate the correlation between the expression of gene and the clinical data and the relationships between modules and the clinical model. The module with the most predictive clinical value was selected for further analyses.

**Fig. 1:**
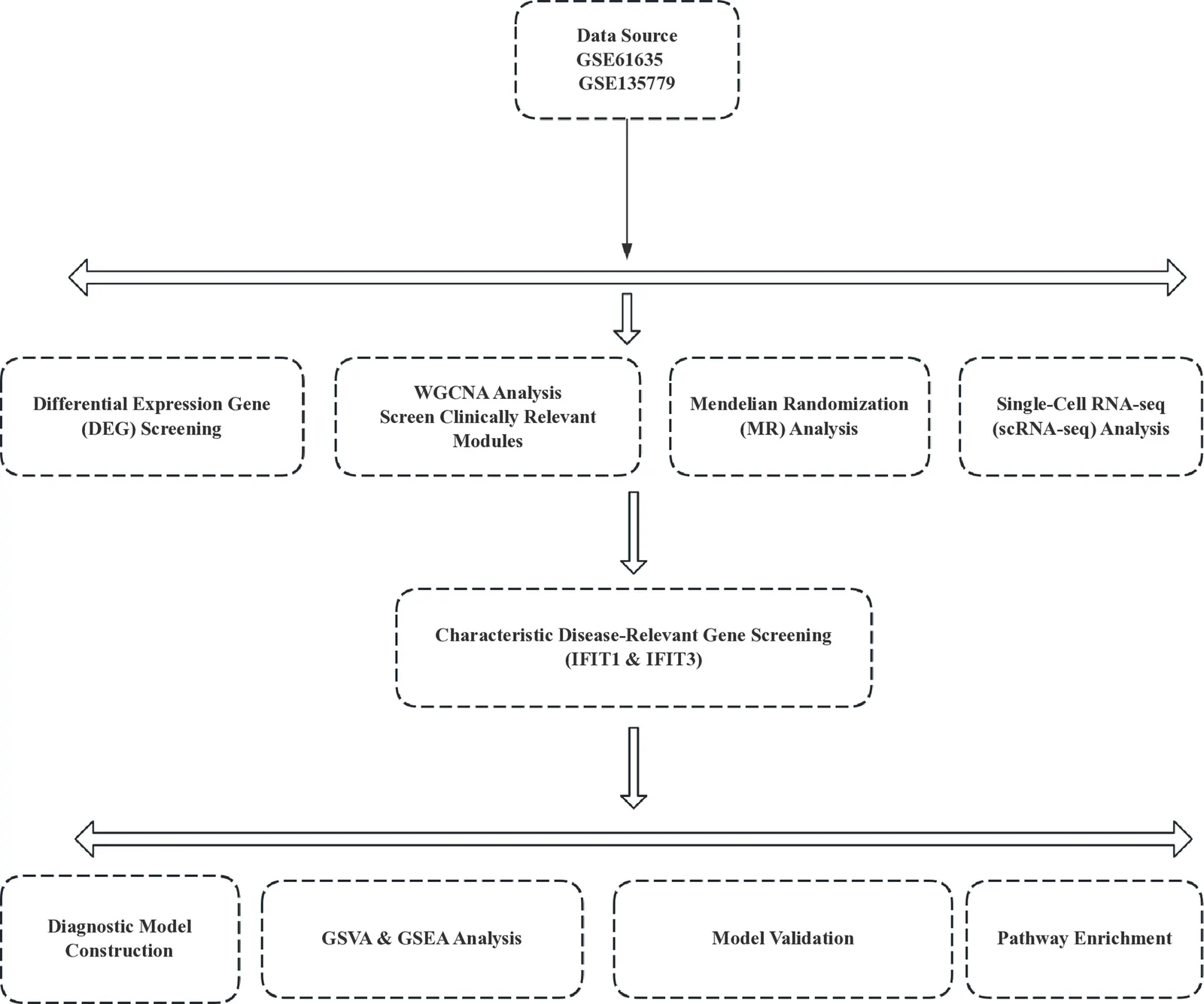
Overview of the Study Workflow. RNA-seq, RNA sequencing; SLE, Systemic Lupus Erythematosus

Finally, single-cell transcriptome data (GSE135779) obtained from the GEO database were analyzed using R and the Seurat package [16]. This included data retrieval, quality control, dimensionality reduction, clustering, and cell type annotation. Marker genes were used to identify subpopulations within each cell cluster. Finally, Mendelian randomization analysis was performed to validate the expression of expression quantitative trait loci (eQTLs) associated with SLE. By analyzing the intersection of four gene sets, we developed a diagnostic model to assess SLE risk. The schematic diagram of the workflow is depicted in Fig. 1.

### Collection of data

This research leveraged the single-cell RNA sequencing data from the GSE135779 dataset, available in the GEO database, to investigate adult and pediatric SLE. The adult cohort comprised 8 SLE patients (aSLE1 to aSLE8) and 6 healthy controls (aHD1 to aHD6), while the pediatric cohort included 33 SLE patients (cSLE1 to cSLE33) and 11 healthy controls (cHD1 to cHD11). Data extraction and analysis were conducted using R and the Seurat package. To ensure the integrity of the data, several quality control procedures were applied: (1) Cells expressing fewer than 200 or more than 4000 genes were discarded; (2) Cells with mitochondrial gene expression representing more than 10% of the total gene expression were also excluded. Following these preprocessing steps, the gene expression data were normalized. For dimensionality reduction, principal component analysis (PCA) was first performed [17], after which dimensionality reduction and cluster visualization were further conducted using uniform manifold approximation and projection (UMAP) [18]. To improve the accuracy of cell subpopulation clustering, t-distributed stochastic neighbor embedding (t-SNE) was also used [19]. Cell subpopulation annotation was conducted on MonacoImmuneData. The training set was GSE61635, and the validation set was GSE5077, with detailed information available in Supplementary Table 1.

### Identification of differentially expressed genes

The “Limma” package was used to identify DEGs in the GSE61635 dataset, with a significance level of *p* < 0.05, and |log2 fold change (FC)| ≥ 0.585. The volcano plot and heatmap of common DEGs were generated using the “ggplot2” and “pheatmap” packages in R [20].

### Weighted gene co-expression network analysis

To establish a weighted co-expression network to standardize gene expression data from the GSE61635 dataset, the “WGCNA” package was used. First, data quality was assessed using the “goodSampleGenes” function to select samples and genes for subsequent analysis. The optimum soft-thresholding power (β) was obtained by using the “pickSoftThreshold” function to build a scale-free topological network, and the expression data were translated to an adjacency matrix. MEs were subsequently computed, and hierarchical clustering of modules was performed to merge modules with similar correlations, thereby yielding a dendrogram. Gene significance (GS) and module significance (MS) were then calculated for all modules to evaluate their correlation with both genetic and clinical characteristics. Furthermore, module membership (MM) was computed for each gene, enabling a more in-depth analysis of the associations between genes within each module and their involvement in specific phenotypes [21].

### Mendelian randomization validation of key gene eQTLs

By intersection of features, we extracted the final intersecting feature genes from IEU Open GWAS database (https://gwas.mrcieu.ac.uk/datasets/), and use the corresponding eQTLs as exposures in MR analysis to investigate the associations between SLE [22]. SLE-related data were obtained from the ebi-a-GCST90011866 dataset, which consists of genome-wide data from 8,431 healthy controls and 4,222 SLE patients. Candidate pathogenic genes associated with SLE were identified, and bidirectional MR analysis was performed to mitigate the issue of reverse causality. For genetic instrument selection, we restricted our focus to single-nucleotide polymorphisms (SNPs) strongly associated with gene expression, applying a stringent significance threshold (*P* < 5 × 10^− 8^). To avoid weak instrument bias, we calculated the F-statistic and excluded SNPs with F ≤ 10. For the primary MR analysis, we employed the inverse-variance weighted (IVW) method [23] and assessed the heterogeneity of instrumental variables using Cochran’s Q test [24], where a value of *P* > 0.05 indicated low heterogeneity. In addition to the IVW method, we applied MR-Egger regression to evaluate potential horizontal pleiotropy, with a statistically significant intercept indicating the presence of pleiotropy.

### Selection of characteristic genes

Finally, the differentially expressed genes, single-cell analysis results, gene modules identified by WGCNA, and key genes identified by Mendelian randomization were intersected to obtain a set of characteristic genes. Further analysis was conducted on the identified gene set to assess its biological and clinical relevance in SLE.

### Construction and validation of the diagnostic model

Utilizing the identified characteristic genes, a diagnostic model was developed to predict the risk of SLE. The model was constructed as a dynamic nomogram, and its clinical utility was evaluated using decision curve analysis (DCA). To further assess prediction accuracy, calibration curves and clinical impact curves were generated. Finally, the discriminative capacity of the model was evaluated using receiver operating characteristic (ROC) curves, with all statistical analyses and curve plotting executed in R software.

### Gene Set Variation Analysis (GSVA) and Gene Set Enrichment Analysis (GSEA)

A non-parametric, unsupervised GSVA approach was adopted to examine the differentially enriched KEGG pathways associated with SLE [25]. Following this, single-gene GSEA was conducted for each characteristic gene using the R package clusterProfiler [26], enabling us to assess differences in KEGG pathway enrichment across gene expression groups.

## Results

### Transcriptomics Reveal the Distinct Cell Composition in SLE

**Fig. 2:**
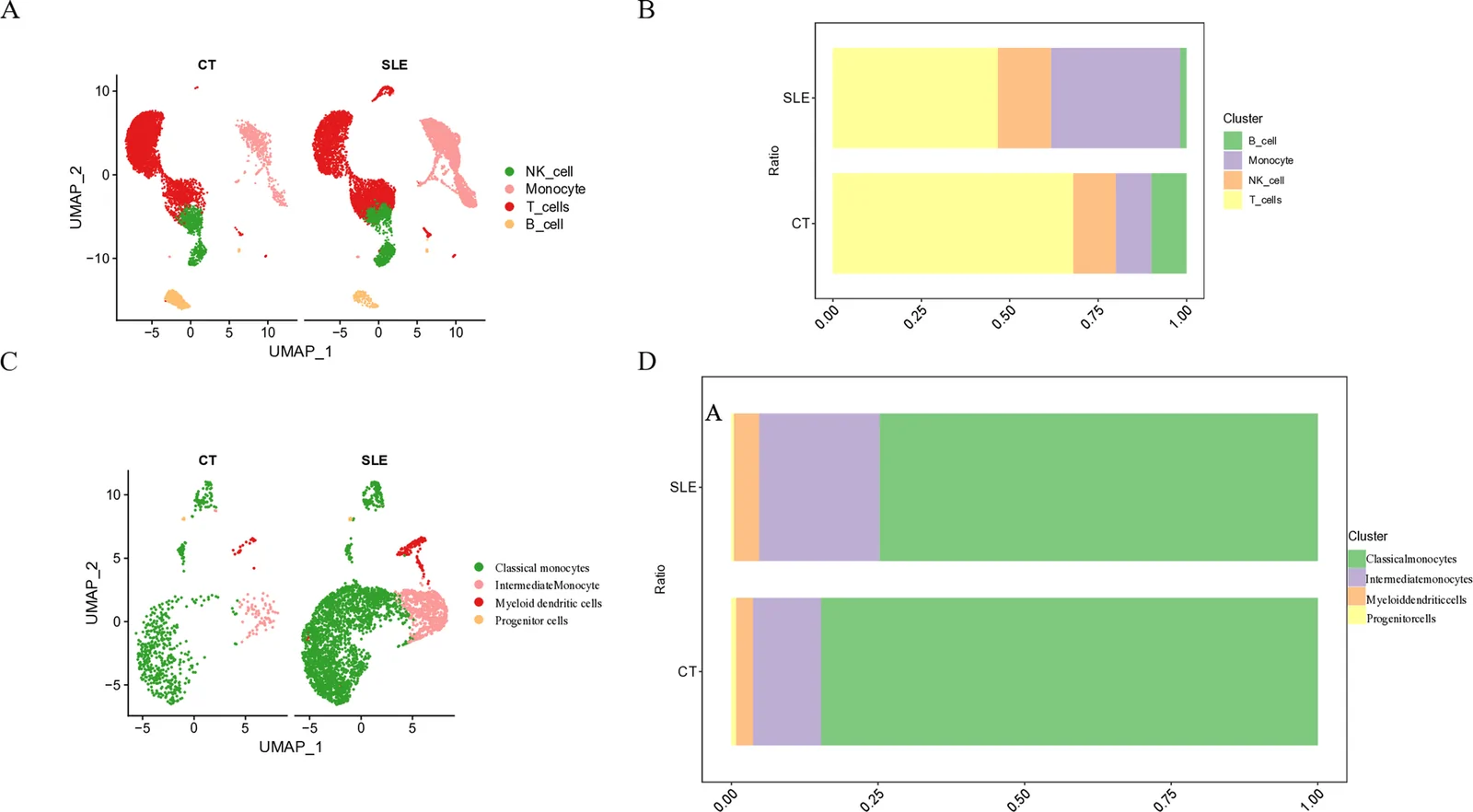
Analysis of single-cell transcriptomic data. **A**, Dimensionality reduction and clustering visualization for SLE samples. **B**, Proportional representation of dimensionality reduction and clustering in SLE samples. **C**, Dimensionality reduction and clustering visualization for monocytes in SLE. **D**, Proportional representation of dimensionality reduction and clustering for SLE monocytes. SLE, Systemic Lupus Erythematosus

Single-cell transcriptomic analysis identified four cell phenotypes, which were annotated in the dimensionality reduction and cell proportion plots based on immune marker gene expression (Fig. 2A and B). Monocytes were strongly enriched in SLE compared with healthy controls, suggesting a major role for monocytes in SLE pathogenesis. Consequently, subset dimensionality reduction and clustering analysis were restricted to the monocyte population (Fig. 2C and D), revealing distinct transcriptomic features between the healthy control and SLE groups.

### Identification of DEGs

**Fig. 3:**
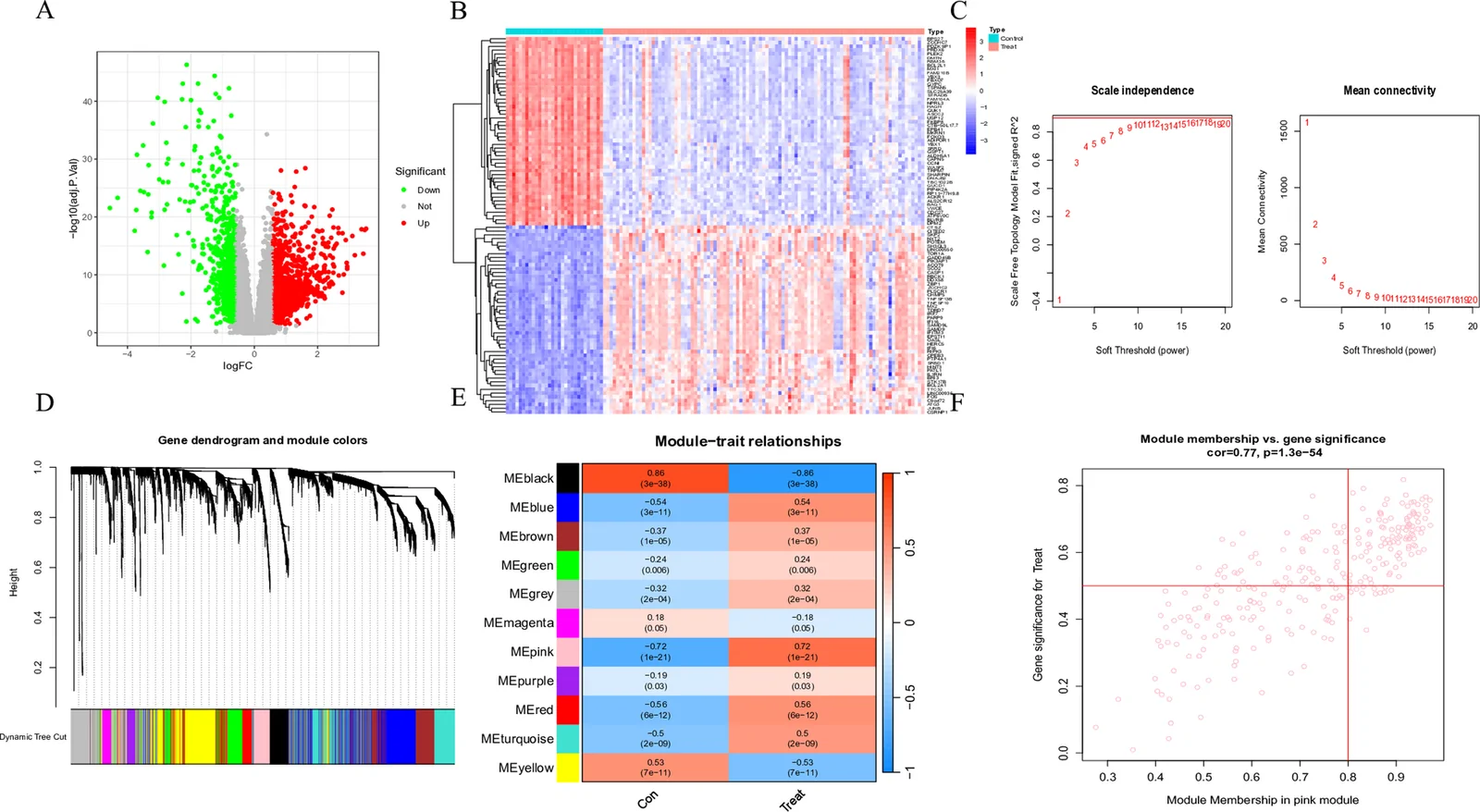
Differentially Expressed Genes and Weighted Gene Co-expression Network Analysis Results of SLE; **A**, Differentially expressed genes in SLE. **B**, Heatmap of differentially expressed genes in SLE. **C**, Relationship between the fit index and soft threshold (left) and between average connectivity and soft threshold (right). **D**, Construction of weighted gene co-expression network analysis (WGCNA) modules in SLE. **E**, Heatmap showing the correlation between the SLE gene module and clinical traits; red indicates positive correlation, while blue indicates negative correlation. **F**, Correlation plot of module membership (MM) versus gene significance (GS) for the module, with a correlation coefficient (cor) of 0.77.SLE, Systemic Lupus Erythematosus

DEGs in the GSE61635 dataset were screened out with the “Limma” package. The volcano plots (Fig. 3A) and heatmaps (Fig. 3B) for most identified DEGs were generated using the “ggplot2” and “pheatmap” packages in R.

### Construction of a co-expression network

Gene co-expression networks were constructed by the WGCNA package. Initially, sample clustering was performed to identify and remove outliers or incomplete data as a quality control measure. To achieve a scale-free topology and effectively segregate gene modules, a scale-free network was constructed using an optimal soft-thresholding power (β = 7), determined via network fit indices (Fig. 3C). A one-step network construction approach was then utilized to generate a highly connected co-expression matrix, followed by the dynamic tree cut method for hierarchical clustering to define gene modules. After merging modules with highly similar expression profiles, 11 distinct gene modules were ultimately identified (Fig. 3D). Finally, the association between modules and clinical traits was visualized using a heatmap (Fig. 3E). Specifically, the pink module, which exhibited a significant correlation between GS and MM (cor = 0.77, *p* = 1.3 × 10^−^⁵⁴, Fig. 3F), was therefore selected as the candidate module for subsequent analysis.

### Selection of characteristic genes

**Fig. 4:**
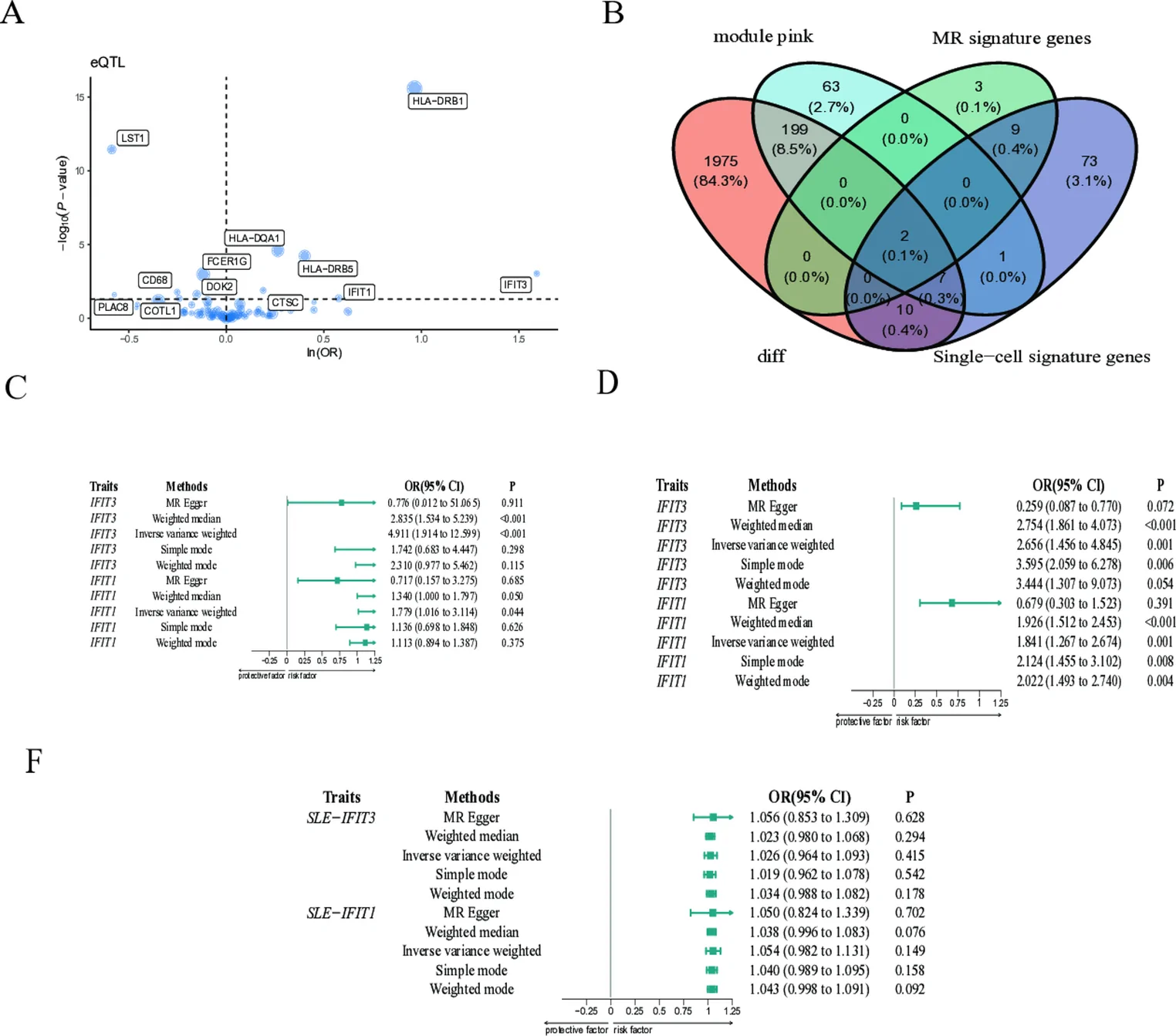
Mendelian correlation analysis of marker genes with SLE. **A**. Mendelian forest plot illustrating the causal relationship between marker genes and SLE. **B**. Intersection plot of characteristic genes. **C**. Validation of causal characteristic genes in an independent SLE cohort. **D**. Reverse MR analysis of characteristic genes with systemic lupus erythematosus; MR: Mendelian randomization; SLE, Systemic Lupus Erythematosus

Screening marker genes of monocytes of patients with SLE and performing MR analysis by using eQTL data to detect their causal relationship to SLE, GWAS data of SLE from the ebi-a-GCST90011866 dataset; reverse MR to eliminate reverse causality, and the results were verified in the independent ebi-a-GCST003156 dataset for robustness. Overall, 12 genes were identified as causal for SLE (Fig. 4A), including *COTL1*,* CTSC*,* IFIT3*,* CD68*,* PLAC8*,* DOK2*,* FCER1G*,* IFIT1*,* HLA-DRB1*,* HLA-DQA1*,* HLA-DRB5*, and *LST1*. By intersecting the MR-confirmed genes with differentially expressed genes, WGCNA pink module genes and single-cell key genes, we ultimately identified two core feature genes: *IFIT1* and *IFIT3* (Fig. 4B). For these two candidate genes, forward MR analysis showed a causal effect (Fig. 4C) and no marked reverse causal effect was observed (Fig. 4F). Replication results of the independent cohort are shown in Fig. 4D. The scatter plots, sensitivity analysis and funnel plot further verified the robustness and accuracy of the MR results (Supplementary Figures S1, S2).

### Construction of an SLE diagnostic model based on feature genes

**Fig. 5:**
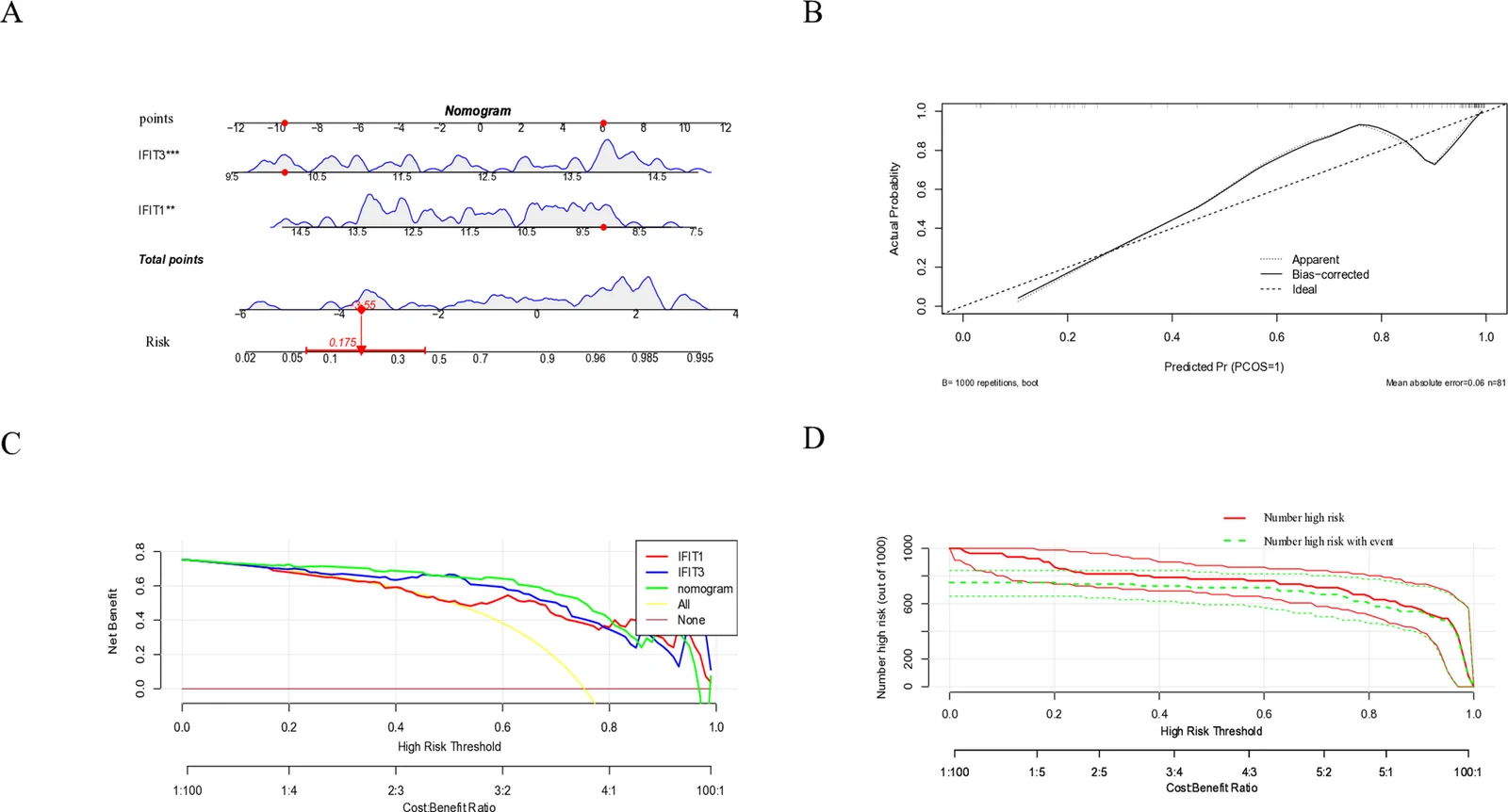
Construction and validation of the diagnostic model for SLE; **A**. Nomogram for SLE; **B**. Calibration curve of the model; **C**. Decision curve analysis; **D**. Clinical impact curve; SLE; Systemic Lupus Erythematosus

An SLE diagnostic nomogram was next constructed utilizing the signature features of *IFIT1* and *IFIT3*. The nomogram, calibration curve, and DCA were generated using R (Fig. 5A and C). The calibration curves showed high consistency between prediction results and actual events. DCA further confirmed the model’s robust predictive effectiveness: the high-risk curve is closer to the high-risk event-related curve at a threshold of 0.2-1. Overall, this finding demonstrates the excellent diagnostic value of the *IFIT1*/*IFIT3*-based nomogram for diagnosing SLE (Fig. 5D).

### Discussion of the specific signaling mechanisms associated with the characteristic genes IFIT1 and IFIT3

Subsequently, the specific signaling pathways associated with *IFIT1* and *IFIT3* were analyzed, and the effects of these candidate genes on disease progression-associated pathways were examined. The results of GSVA suggested that high *IFIT1* expression was associated with the pathways of mismatch repair, nucleotide excision repair, valine, leucine and isoleucine biosynthesis, RNA polymerase, terpenoid backbone biosynthesis, aminoacyl-tRNA biosynthesis, N-glycan biosynthesis, valine, leucine and isoleucine degradation and circadian rhythm in mammals (Supplementary Figures S3A). High *IFIT3* expression was mainly associated with pathways such as primary bile acid biosynthesis, limonene and pinene degradation, glycosaminoglycan degradation and glycosphingolipid biosynthesis (Supplementary Figures S3B). GSEA results also showed that high *IFIT1* expression was enriched in the RIG-like receptor signaling pathway and the cytosolic DNA sensing pathway (Supplementary Figures S3C). On the other hand, high *IFIT3* expression was almost exclusively enriched in pathways related to the cell cycle, chemokine signaling, cytokine-cytokine receptor interaction, NOD-like receptor signaling, RIG-I-like receptor signaling, and Toll-like receptor signaling (Supplementary Figures S3D). *IFIT1* was related to DNA repair, amino acid metabolism, and circadian rhythm, which participate in genomic stability and immune regulation. In contrast, *IFIT3* affected the cell cycle, cytokine signaling and immune pathways, such as the RIG-I-like receptor signaling pathway, in the immune response and the progression of SLE. Taken together, the above findings suggest that *IFIT1* and *IFIT3* are primarily involved in immune regulation, metabolism, and genomic stability and are potential therapeutic targets for SLE.

### Single-Cell RNA sequencing analysis of characteristic genes

**Fig. 6:**
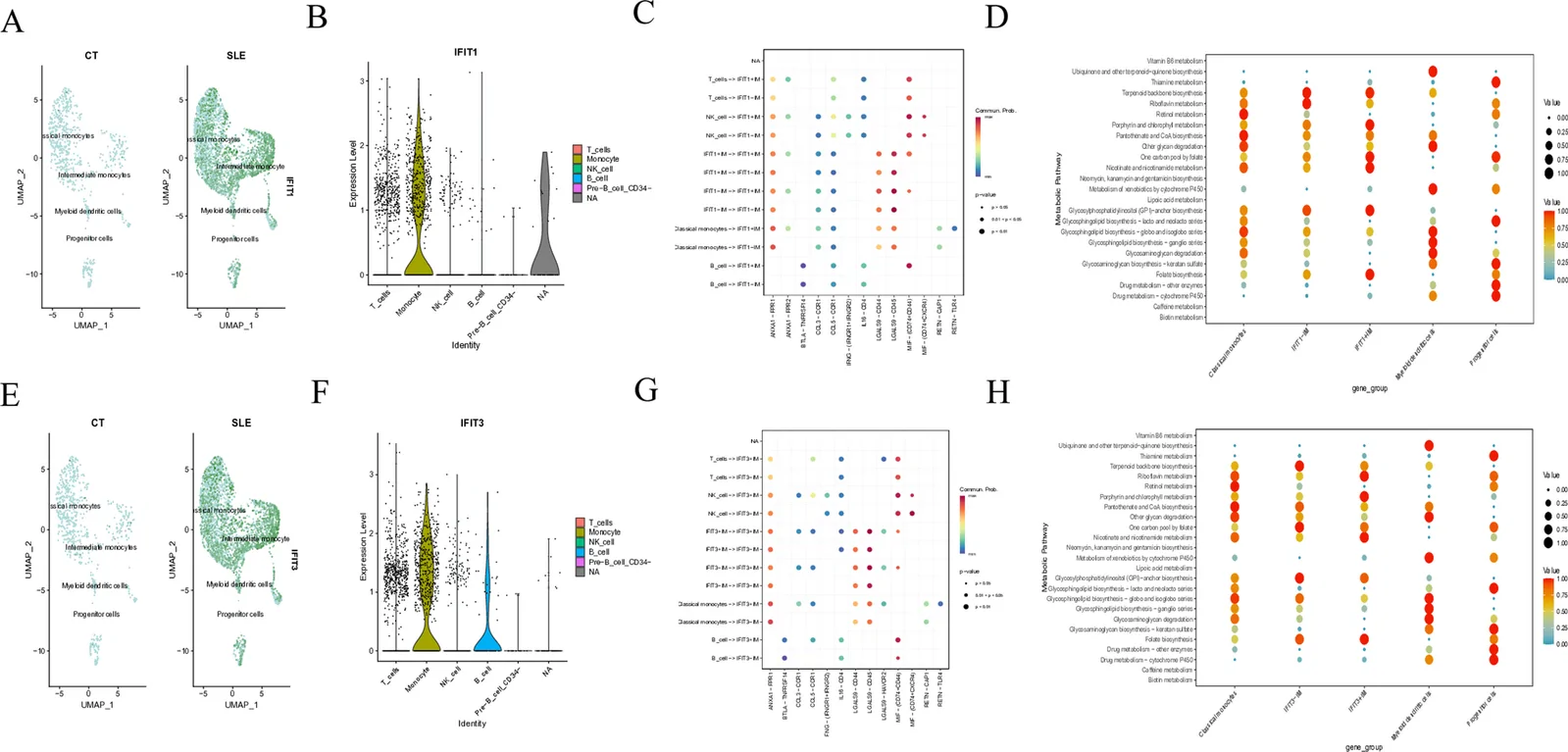
Transcriptomic Analysis of Characteristic Genes. **A**. UMAP visualization of IFIT1 across different cell types. **B**. Expression of IFIT1 in various cell types. **C** Proportional representation of cell communication between IFIT1-positive and IFIT1-negative groups. **D**. Comparative metabolite analysis between IFIT1-positive and IFIT1-negative groups. **E**. UMAP visualization of IFIT3 across different cell types. **F**. Expression of IFIT3 in various cell types. **G**. Proportional representation of cell communication between IFIT3-positive and IFIT3-negative groups. **H**. Comparative metabolite analysis between IFIT3-positive and IFIT3-negative groups

Our study established a causal relationship between the *IFIT1* and *IFIT3* genes and SLE. We assessed the expression levels of these 2 genes at the cellular level in monocytes and found that they were strongly associated with SLE risk, with significantly higher levels in SLE patients than in healthy controls (Fig. 6A, E). Both *IFIT1* and *IFIT3* were also highly expressed in monocytes and T cells (Fig. 6B, F). The cell communication and metabolic pathways between intermediate monocytes in the IFIT1- and IFIT3-positive groups and the IFIT1- and IFIT3-negative groups were further analyzed: the IFIT1-positive intermediate monocyte group showed increases in ANXA1-FPR2 and MIF-(CD74 + CD44) compared with the IFIT1-negative intermediate monocyte group (Fig. 6C). At the metabolic level, differences were observed in some pathways, including terpenoid backbone biosynthesis, riboflavin metabolism, porphyrin and chlorophyll metabolism, one-carbon pool via folate, nicotinate and nicotinamide metabolism and folate biosynthesis (Fig. 6D). Similarly, when comparing the intermediate monocyte groups by IFIT3 expression, the IFIT3-positive group exhibited enhanced CCL5-CCR1, LGALS9-HAVCR2, and MIF-(CD74 + CD44) signaling pathways compared with the IFIT3-negative group (Fig. 6G). At the metabolism level, differences were observed in pathways such as terpenoid backbone biosynthesis, riboflavin metabolism, porphyrin and chlorophyll metabolism, one-carbon pool by folate, nicotinate and nicotinamide metabolism and glycosphingolipid biosynthesis (globo and isoglobo series) (Fig. 6H). IFIT1 promoted immune cell migration and activation via the ANXA1-FPR2 and MIF-CD74 + CD44 pathways, and enhanced immune cell function through metabolic pathways, including riboflavin and folate metabolism. IFIT3, on the other hand, regulated immunity by upregulating the CCL5-CCR1 and LGALS9-HAVCR2 signaling pathways, and by regulating the cell cycle and immune responses; it also regulated terpenoid and glycosphingolipid synthesis. Taken together, these data demonstrate that IFIT1 and IFIT3 are actively involved in the immune dysregulation of SLE, highlighting their potential as promising diagnostic biomarkers and therapeutic targets for the disease.

### Evaluation of nomogram model performance using training and validation datasets

Next, the performance of the nomogram model established with the two shared feature genes identified was measured by plotting ROC curves with respective AUC values to evaluate the performance of the model in diagnosing SLE. Plotting ROC curve in GSE61635 training set, the AUC values of IFIT1 and IFIT3 were 0.933 and 0.960 respectively (Supplementary Figures S4A). Besides, the nomogram model based on the two shared feature genes achieved high accuracy, with an AUC of 0.974 (95% CI: 0.946 to 0.996) (Supplementary Figures S4B). In the GSE50772 validation set, the AUC values of *IFIT1* and *IFIT3* were 0.842 and 0.906, respectively (Supplementary Figures S4C). The nomogram model exhibited strong accuracy in the validation set, with an AUC value of 0.912 (95% CI: 0.816 to 0.984) (Supplementary Figures S4D). Overall, these findings indicate that *IFIT1*, *IFIT3* and the constructed nomogram model are potentially effective biomarkers for identifying and diagnosing SLE.

## Discussion

This study integrated bulk tissue and scRNA-seq data using multiple bioinformatics approaches to comprehensively characterize the molecular mechanisms of SLE. Through differential gene analysis, WGCNA, MR and GSEA, a series of genes involved in the pathogenesis of SLE were identified. Among these, *IFIT1* and *IFIT3* were mainly involved in immune disruption and the development of pathological processes in SLE. These findings make *IFIT1* and *IFIT3* potential biomarkers for identifying and diagnosing SLE, offering a new perspective and a potential strategy for early identification and individualized treatment.

Type I interferons, such as IFN-α, play an essential role in eliciting immune activation in SLE, and type I interferon activation is a major feature of disease progression [24, 27, 28]. Our study observed increased expression of *IFIT1* and *IFIT3*, two genes that are related to the type I interferon signaling pathway, in patients with SLE. This finding suggests that these genes are important for immune regulation and the development of SLE [29]. Specifically, elevated expression of *IFIT1* and *IFIT3* enhances immune cells’ responses to external stimuli and leads to immune system imbalance, inflammation and injury in SLE. Mechanistically, *IFIT1* and *IFIT3* contribute to SLE development through several connected mechanisms: (1) increasing antigen-presenting activity of dendritic cells which, in turn, activate autoreactive T cells and B cells [30]; (2) inducing secretion of pro-inflammatory cytokines such as IL-6 and TNF-α, and further augmenting systemic inflammation [31]; and (3) inducing autoantibody secretion such as anti-double-stranded DNA (anti-dsDNA) antibodies, participating in the immune tolerance disorder leading to attack of self-tissue [32, 33]. Moreover, overexpression of *IFIT1* and *IFIT3* may lead to positive feedback in the immune system and also enhance immune activation, thereby resulting in SLE’s typical chronic immune activation and inflammation [34, 35]. Thus, this upregulation is one hallmark of the chronic inflammatory state typically observed in SLE. Single-cell analysis demonstrated marked upregulation of *IFIT1* and *IFIT3* expression in monocytes and T cells from SLE patients, and this elevated expression in monocytes and T cells from SLE patients is related to immune cell activation. Monocytes are pivotal players in SLE immunopathology, contributing to immune responses, antigen presentation and pro-inflammatory signaling [36, 37]. IFIT1 and IFIT3-positive monocytes exhibited altered immune cell communication and metabolism, such as ANXA1-FPR2, MIF-(CD74 + CD44) and others involved in inflammation and immune cell trafficking; ANXA1 is a pivotal regulator of immune cell trafficking [38] and FPR2 is critical for monocyte recruitment. Similarly [39], MIF acts via CD74 and CD44 to induce immune activation and expansion of inflammation in SLE. Similarly, these results highlight the role of IFIT1 in increasing immune cell recruitment and activation in SLE. In parallel, IFIT1-positive monocytes are activated towards the CCL5-CCR1, LGALS9-HAVCR2 and MIF-(CD74 + CD44) pathways, all of which are relevant to immune cell chemotaxis, activation and cytokine secretion. Altered metabolic pathways in these cells also include riboflavin metabolism, porphyrin metabolism and terpenoid biosynthesis, indicating that disordered cellular energy and immune cell metabolism are fundamental aspects of SLE pathogenesis. These findings collectively demonstrate the importance of immune cell metabolism for driving immune cell dysfunction in SLE.

Our analysis further identified dysregulated pathways, including riboflavin, porphyrin and chlorophyll metabolism, in IFIT1- and IFIT3-positive monocytes, all of which are related to cellular energy production, immune response regulation and immune cell functions. Metabolic reprogramming in immune cells is a known phenomenon in inflammatory diseases, including SLE, and is associated with changes in cellular energetics, providing fuel to enhance immune activation. This imbalance may be the driver of the chronic immune activation and inflammation in SLE. Studies investigating the relationship between immune cell metabolism and gene expression can uncover new therapeutic targets to restore metabolic balance and immune homeostasis in SLE. Targeting immune cell metabolic pathways could be an interesting approach to influence immune responses and dampen disease activity.

Furthermore, GSEA and GSVA revealed the specific molecular mechanisms by which *IFIT1* and *IFIT3* contribute to the onset of SLE. *IFIT1* was enriched in pathways related to genetic stability, immunity regulation and cell cycle progression. Specifically, *IFIT1* was involved in DNA repair, nucleotide excision repair, and circadian rhythm regulation, pathways important for guaranteeing genomic stability of immune cells and for immune regulatory responses [40]. Abnormalities in these pathways can cause genomic instability in immune cells and activate abnormal immune responses, leading to the development of SLE. In contrast, *IFIT3* was enriched in pathways involved in activation of immune cells and immune autoimmune responses, such as chemokine signaling pathway, TLR signaling pathway and RIG-I-like receptor signaling pathway [41, 42]. Indeed, these pathways are important in the generation of innate immune response, antiviral responses, and inflammation. Upregulation of *IFIT3* activates these pathways, resulting in excessive immune activation and tissue damage in SLE.

In addition to its mechanistic explanation, our study developed a diagnostic model based on the expression levels of *IFIT1* and *IFIT3*, using the dynamic nomogram method. Validated across multiple datasets (including GSE61635 and GSE50772), our model was robust and demonstrated strong predictive performance. In the training set (GSE61635), *IFIT1* and *IFIT3* exhibited very high AUC values (0.933 and 0.960, respectively), and the combined model yielded an AUC value of 0.974. In the test set (GSE50772), *IFIT1* and *IFIT3* also exhibited high AUC values (0.842 and 0.906, respectively), and the model achieved an AUC of 0.912, further confirming the model’s robustness. Both DCA and calibration curve analysis demonstrated the potential clinical practicability of this diagnostic model, especially for early diagnosis of SLE and prognosis stratification. Importantly, our model also offers a useful tool for identifying high-risk individuals and providing individualized treatment strategies for SLE. *IFIT1* and *IFIT3* hold great promise as diagnostic biomarkers for SLE and will enable early, easy identification of the disease (particularly in patients with discrete or early-stage symptoms). Meanwhile, the diagnostic model based on *IFIT1* and *IFIT3* also provides not only insight into disease activity but also facilitates individualized treatment for patients with SLE. Next steps for future research include developing new strategies to regulate *IFIT1* and *IFIT3* for targeted treatment and restoring immune homeostasis to prevent and ameliorate immune injury in patients with SLE. Combining data (such as proteomics and metabolomics) will further elucidate the mechanisms underlying SLE pathogenesis and facilitate the development of more effective diagnostic methods and therapeutic approaches.

### Strengths and Limitations

Our study identified IFIT1 and IFIT3 as core hub genes, demonstrating their cell-type-specific upregulation in monocytes and T cells from SLE patients at single-cell resolution. Unlike existing multi-gene interferon scores, the *IFIT1*/*IFIT3* signature offers specific advantages and translational feasibility. In contrast to the traditionally used interferon scoring, the two-gene model can be analyzed quickly and cheaply by qPCR and flow cytometry, enabling convenient use in clinical settings. Through the integration of bulk and single-cell RNA-seq analyses, we further revealed unique changes in cell communication, riboflavin and porphyrin metabolism and ANXA1-FPR2 signaling in IFIT1/IFIT3-positive monocytes. These previously unrecognized molecular and metabolic features of SLE cannot be captured by a conventional interferon score, and the results of the current study provide new insights into its pathogenesis. Mechanically, upregulation of *IFIT1* and *IFIT3* modulates innate and adaptive immunity and induces inflammation, immune activation and autoantibody production in SLE. Using this constructed two-gene model for early SLE diagnosis, we have accurately diagnosed early-stage SLE. Interestingly, both *IFIT1* and *IFIT3* exhibit contrasting functions: while *IFIT1* plays divergent roles in DNA repair and circadian rhythm, *IFIT3* exerts opposing effects in the cell cycle and chemokine signaling. Such functional independence might help differentiate SLE subtypes and predict treatment responses, supporting its potential for prognosis and clinical classification. Several limitations of this study should be acknowledged. All results were based on retrospective public datasets, without prospective cohort validation or direct comparisons of performance with classic interferon scoring systems. The bases of such transcriptomic data could not reflect the multi-layered regulation of the proteome and metabolome in SLE immune dysfunction. Future studies will validate this signature in large, multicentre, prospective cohorts of SLE patients, autoimmune disease controls and healthy individuals. Its performance will be further compared against existing interferon scores, with data integrated to provide a comprehensive interpretation of SLE immunometabolic pathology and to further fine-tune the underlying regulatory mechanisms.

## Conclusions

Our analyses validated enhanced expression of the core ISGs *IFIT1* and *IFIT3* in monocytes and T cells of SLE patients. By regulating immune activation and promoting metabolic reprogramming, these two genes can induce SLE and serve as excellent diagnostic biomarkers. The *IFIT1*/*IFIT3* two-gene model demonstrates high diagnostic accuracy, facilitating early screening for SLE patients and individualized therapy. Compared with the conventional interferon scoring system, this simple dual-gene signature is more conducive to clinical application and can serve as a complement, not a replacement, for the corresponding biomarker. Further validation in larger clinical populations, along with related work on corresponding targeted therapeutic applications, is warranted.

## Supplementary Information

Below is the link to the electronic supplementary material.


Supplementary Material 1



Supplementary Material 2



Supplementary Material 3



Supplementary Material 4



Supplementary Material 5


## Data Availability

All datasets used in this study are accessible for download from the Gene Expression Omnibus (https://www.ncbi.nlm.nih.gov/geo/) and the IEU Open GWAS project database (https://gwas.mrcieu.ac.uk/datasets/).
