# Supplementary material for: Role of IFIT1 and IFIT3 in systemic lupus erythematosus: modeling a diagnosis and exploring immune regulation
Source: Clin Exp Med. 2026 Jul 24;26(1):313. doi: 10.1007/s10238-026-02265-6 (PMC13534192; doi:10.1007/s10238-026-02265-6)
Supplement: Supplementary file 1 [file 10238_2026_2265_MOESM2_ESM.pdf]

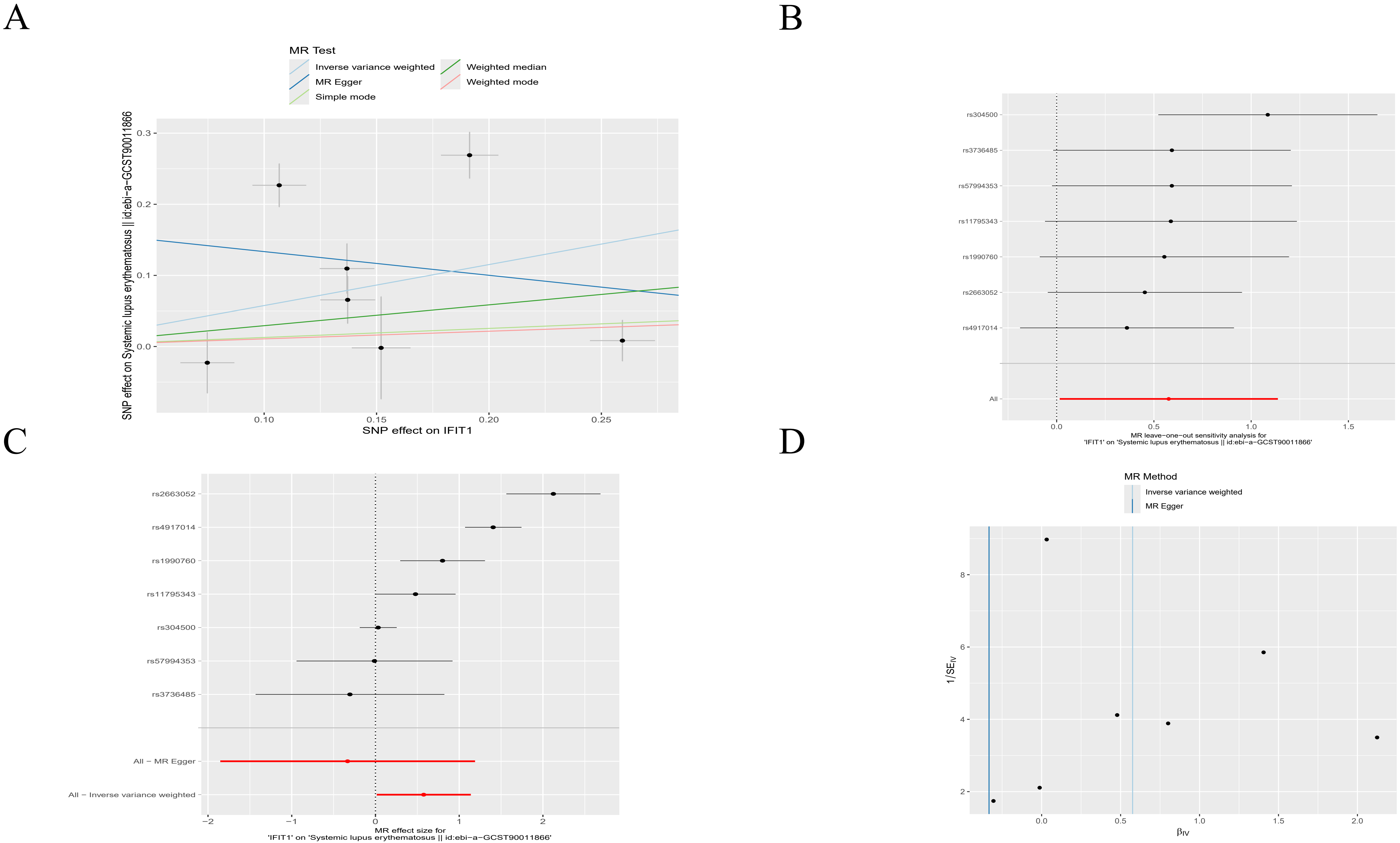

A
MR Test
Inverse variance weighted
MR Egger
Simple mode
Weighted median
Weighted mode
SNP effect on Systemic lupus erythematosus || id:ebi-a-GCST90011866
SNP effect on IFIT1
B
rs304500
rs3736485
rs57994353
rs11795343
rs1990760
rs2663052
rs4917014
All
MR leave-one-out sensitivity analysis for
'IFIT1' on 'Systemic lupus erythematosus || id:ebi-a-GCST90011866'
C
rs2663052
rs4917014
rs1990760
rs11795343
rs304500
rs57994353
rs3736485
All - MR Egger
All - Inverse variance weighted
MR effect size for
'IFIT1' on 'Systemic lupus erythematosus || id:ebi-a-GCST90011866'
D
MR Method
Inverse variance weighted
MR Egger
1/SE_IV
β_IV
