# Supplementary material for: Role of IFIT1 and IFIT3 in systemic lupus erythematosus: modeling a diagnosis and exploring immune regulation
Source: Clin Exp Med. 2026 Jul 24;26(1):313. doi: 10.1007/s10238-026-02265-6 (PMC13534192; doi:10.1007/s10238-026-02265-6)
Supplement: Supplementary file 2 [file 10238_2026_2265_MOESM3_ESM.pdf]

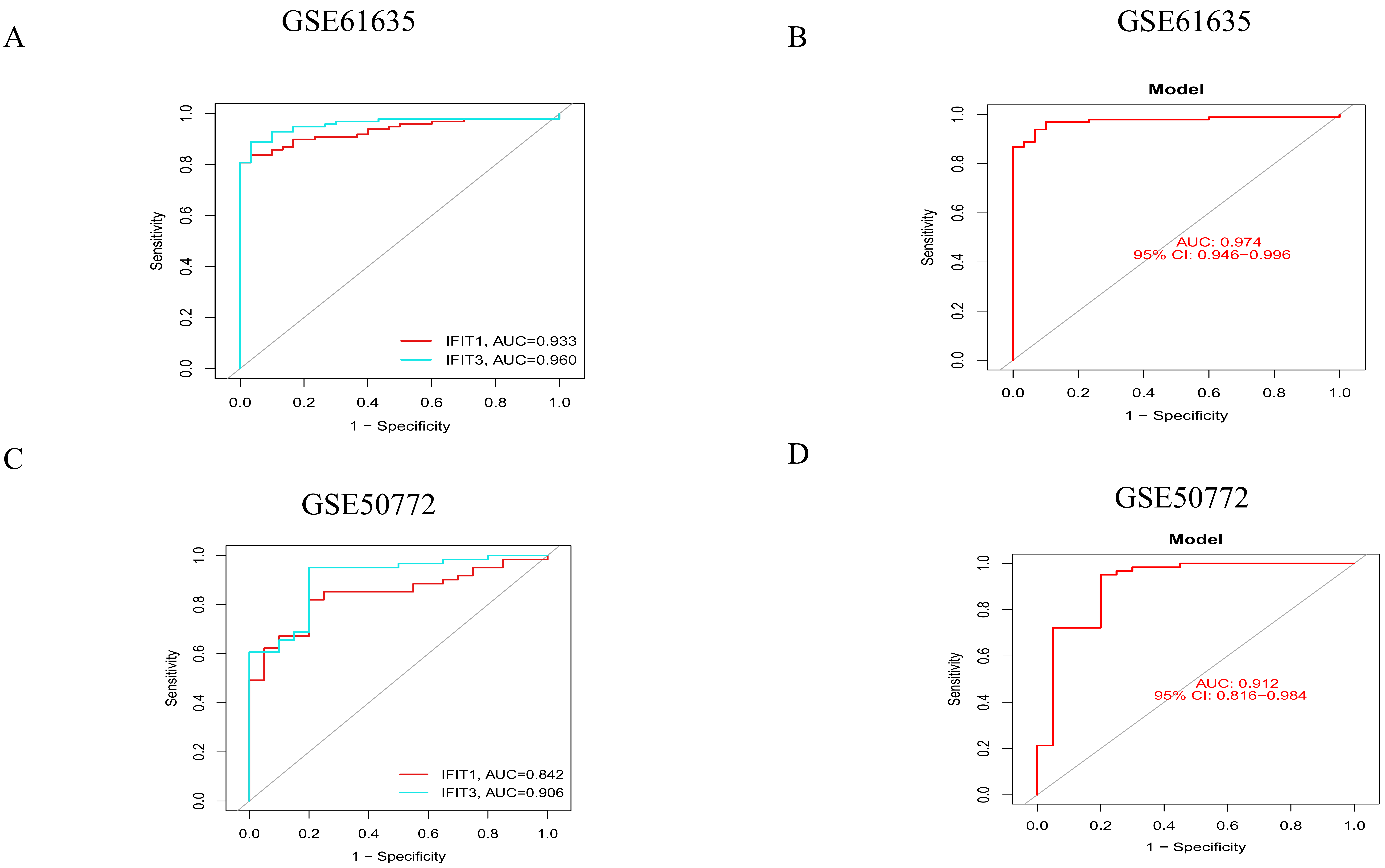

A
GSE61635
Sensitivity
1 − Specificity
IFIT1, AUC=0.933
IFIT3, AUC=0.960
B
GSE61635
Model
Sensitivity
1 − Specificity
AUC: 0.974
95% CI: 0.946−0.996
C
GSE50772
Sensitivity
1 − Specificity
IFIT1, AUC=0.842
IFIT3, AUC=0.906
D
GSE50772
Model
Sensitivity
1 − Specificity
AUC: 0.912
95% CI: 0.816−0.984
