# Supplementary material for: Role of IFIT1 and IFIT3 in systemic lupus erythematosus: modeling a diagnosis and exploring immune regulation
Source: Clin Exp Med. 2026 Jul 24;26(1):313. doi: 10.1007/s10238-026-02265-6 (PMC13534192; doi:10.1007/s10238-026-02265-6)
Supplement: Supplementary file 3 [file 10238_2026_2265_MOESM4_ESM.pdf]

A

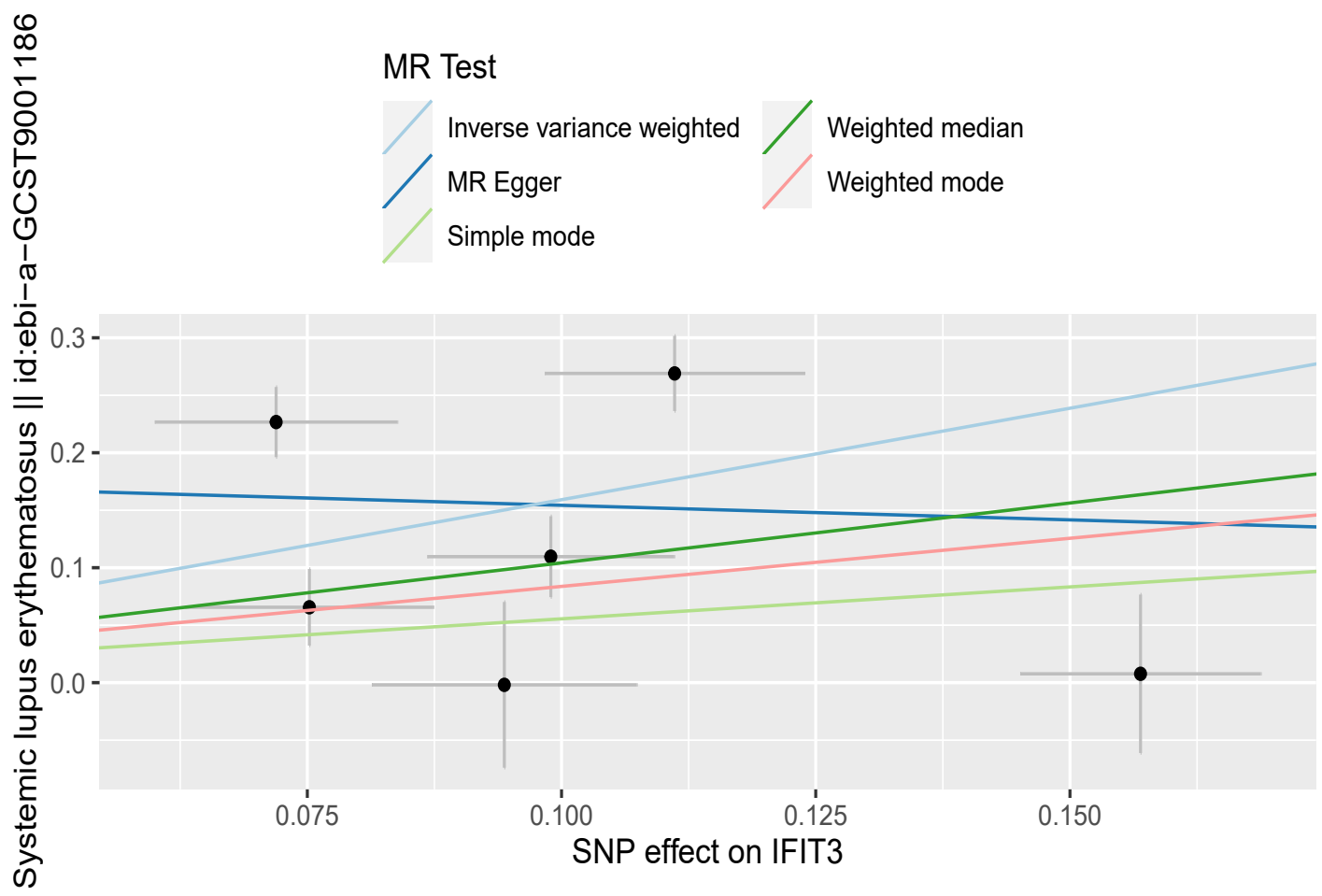
MR Test
Inverse variance weighted
MR Egger
Simple mode
Weighted median
Weighted mode
Systemic lupus erythematosus || id:ebi-a-GCST9001186
0.3
0.2
0.1
0.0
0.075
0.100
0.125
0.150
SNP effect on IFIT3

B

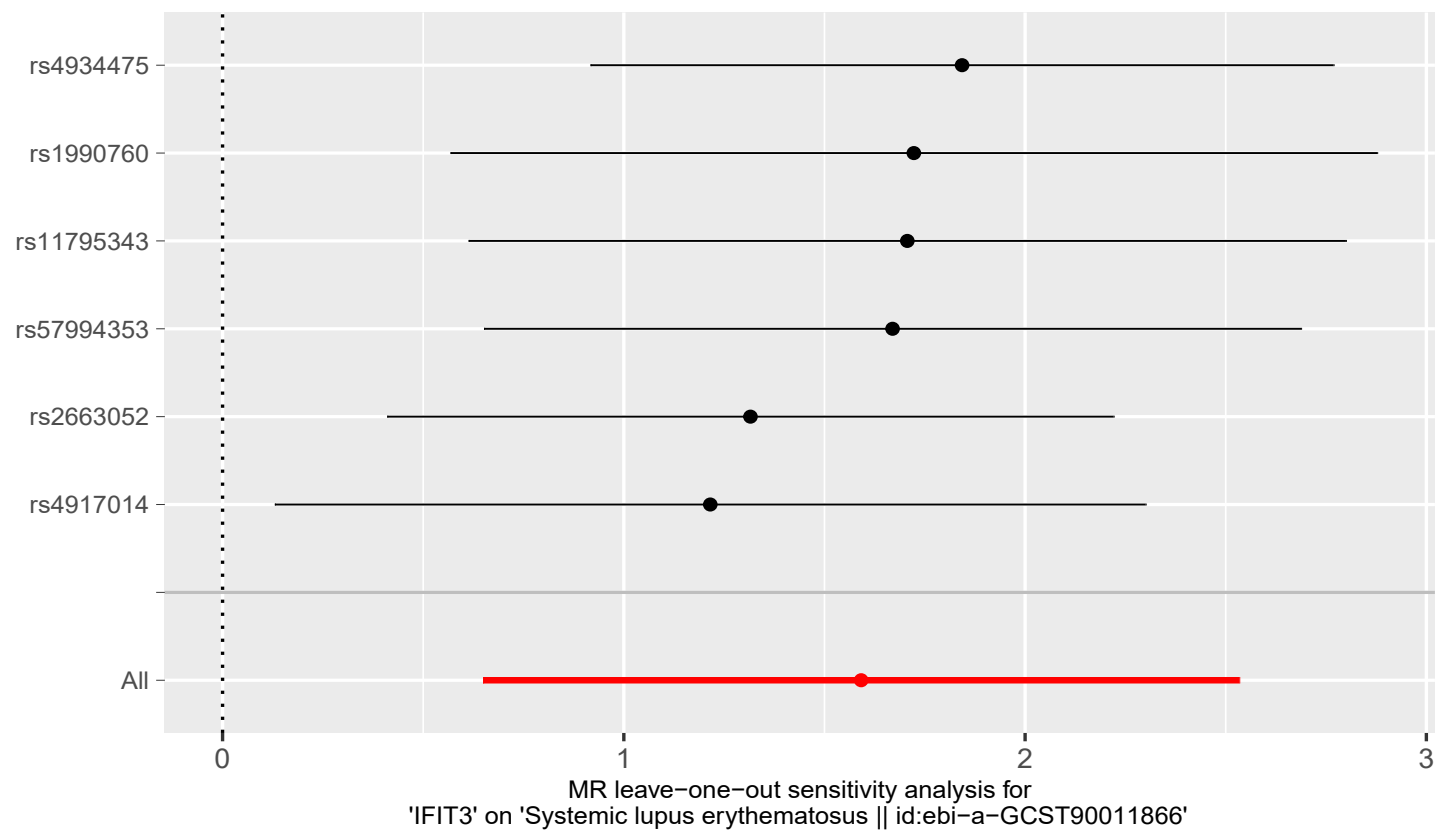
rs4934475
rs1990760
rs11795343
rs57994353
rs2663052
rs4917014
All
0
1
2
3
MR leave-one-out sensitivity analysis for
'IFIT3' on 'Systemic lupus erythematosus || id:ebi-a-GCST90011866'

C

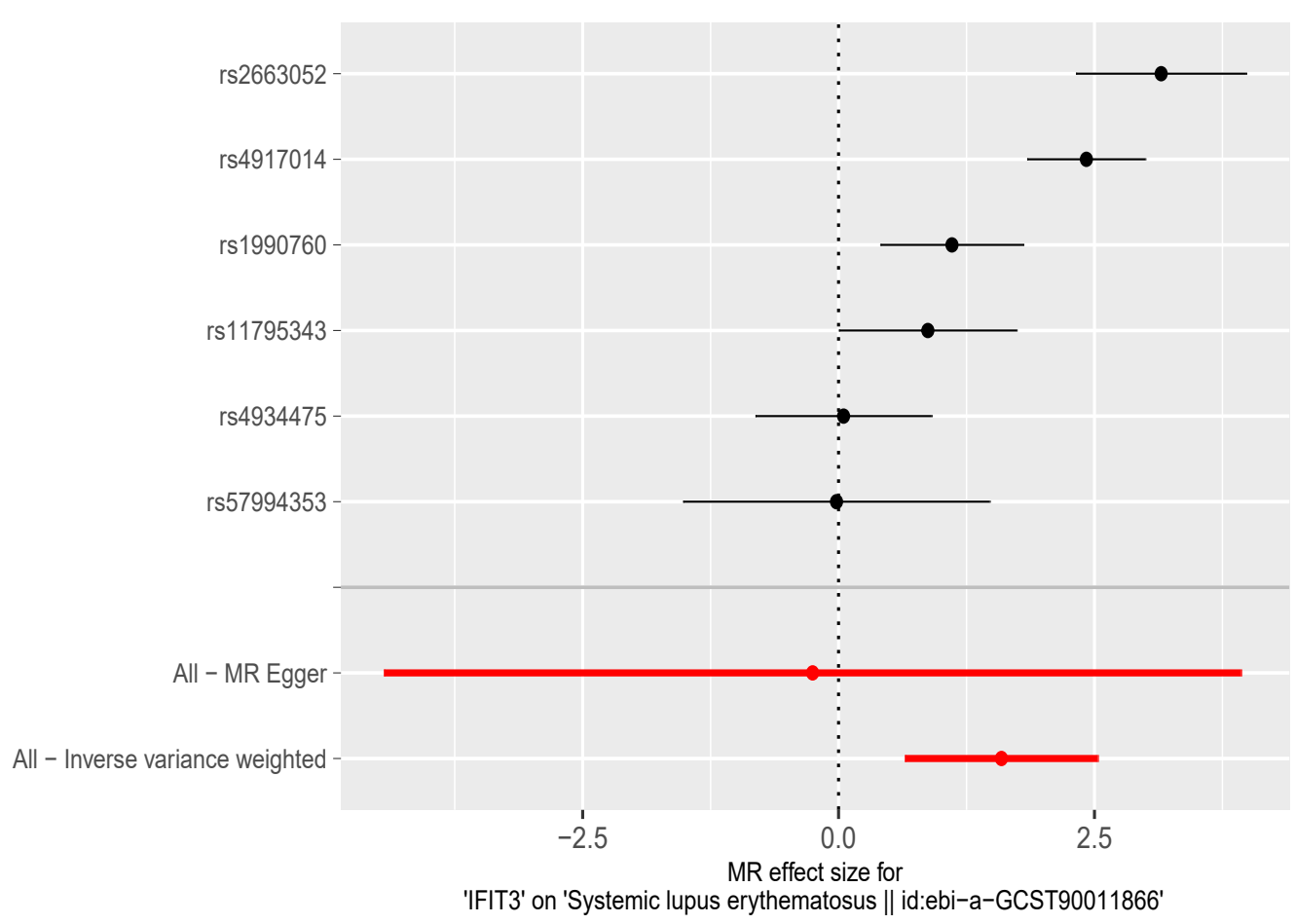
rs2663052
rs4917014
rs1990760
rs11795343
rs4934475
rs57994353
All − MR Egger
All − Inverse variance weighted
-2.5
0.0
2.5
MR effect size for
'IFIT3' on 'Systemic lupus erythematosus || id:ebi-a-GCST90011866'

D

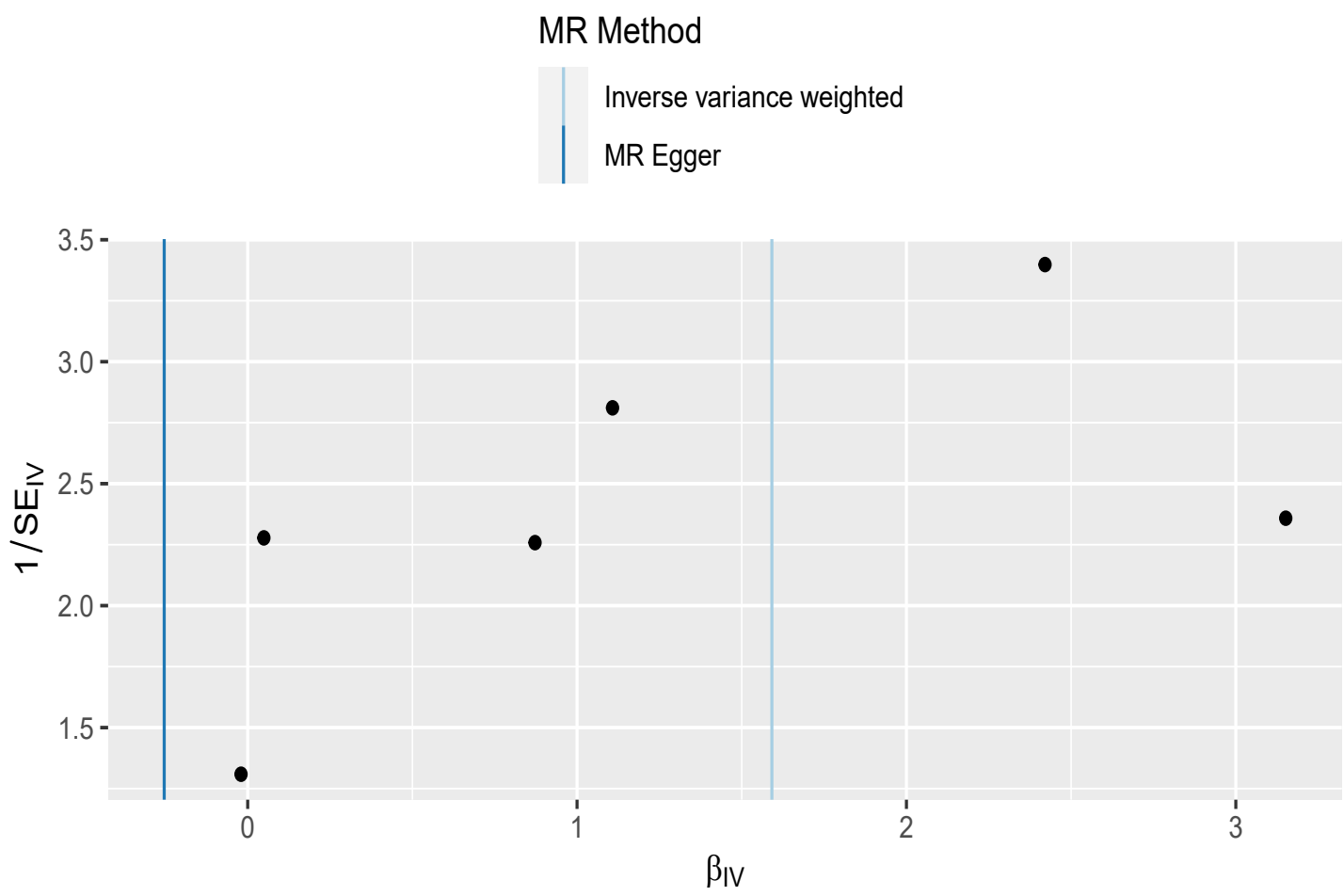
MR Method
Inverse variance weighted
MR Egger
3.5
3.0
2.5
2.0
1.5
1/SE_IV
0
1
2
3
β_IV
