# Supplementary material for: Role of IFIT1 and IFIT3 in systemic lupus erythematosus: modeling a diagnosis and exploring immune regulation
Source: Clin Exp Med. 2026 Jul 24;26(1):313. doi: 10.1007/s10238-026-02265-6 (PMC13534192; doi:10.1007/s10238-026-02265-6)
Supplement: Supplementary file 4 [file 10238_2026_2265_MOESM5_ESM.pdf]

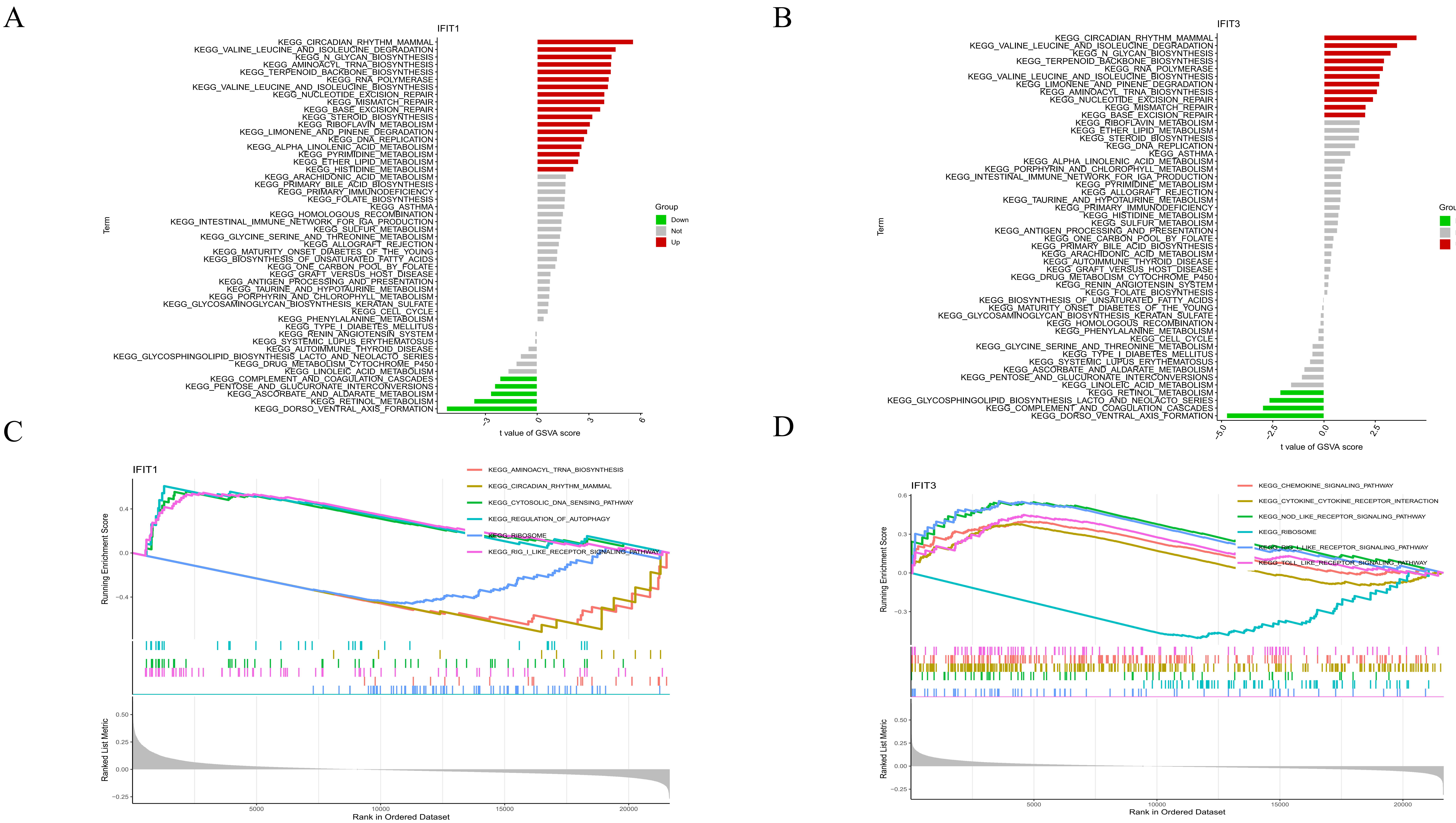

A
IFIT1
KEGG_CIRCADIAN_RHYTHM_MAMMAL
KEGG_VALINE_LEUCINE_AND_ISOLEUCINE_DEGRADATION
KEGG_N_GLYCAN_BIOSYNTHESIS
KEGG_AMINOACYL_TRNA_BIOSYNTHESIS
KEGG_TERPENOID_BACKBONE_BIOSYNTHESIS
KEGG_RNA_POLYMERASE
KEGG_VALINE_LEUCINE_AND_ISOLEUCINE_BIOSYNTHESIS
KEGG_NUCLEOTIDE_EXCISION_REPAIR
KEGG_MISMATCH_REPAIR
KEGG_BASE_EXCISION_REPAIR
KEGG_STEROID_BIOSYNTHESIS
KEGG_RIBOFLAVIN_METABOLISM
KEGG_LIMONENE_AND_PINENE_DEGRADATION
KEGG_DNA_REPLICATION
KEGG_ALPHA_LINOLENIC_ACID_METABOLISM
KEGG_PYRIMIDINE_METABOLISM
KEGG_ETHER_LIPID_METABOLISM
KEGG_HISTIDINE_METABOLISM
KEGG_ARACHIDONIC_ACID_METABOLISM
KEGG_PRIMARY_BILE_ACID_BIOSYNTHESIS
KEGG_PRIMARY_IMMUNODEFICIENCY
KEGG_FOLATE_BIOSYNTHESIS
KEGG_ASTHMA
KEGG_HOMOLOGOUS_RECOMBINATION
KEGG_INTESTINAL_IMMUNE_NETWORK_FOR_IGA_PRODUCTION
KEGG_SULFUR_METABOLISM
KEGG_GLYCINE_SERINE_AND_THREONINE_METABOLISM
KEGG_ALLOGRAFT_REJECTION
KEGG_MATURITY_ONSET_DIABETES_OF_THE_YOUNG
KEGG_BIOSYNTHESIS_OF_UNSATURATED_FATTY_ACIDS
KEGG_ONE_CARBON_POOL_BY_FOLATE
KEGG_GRAFT_VERSUS_HOST_DISEASE
KEGG_ANTIGEN_PROCESSING_AND_PRESENTATION
KEGG_TAURINE_AND_HYPOTAURINE_METABOLISM
KEGG_PORPHYRIN_AND_CHLOROPHYLL_METABOLISM
KEGG_GLYCOSAMINOGLYCAN_BIOSYNTHESIS_KERATAN_SULFATE
KEGG_CELL_CYCLE
KEGG_PHENYLALANINE_METABOLISM
KEGG_TYPE_I_DIABETES_MELLITUS
KEGG_RENIN_ANGIOTENSIN_SYSTEM
KEGG_SYSTEMIC_LUPUS_ERYTHEMATOSUS
KEGG_AUTOIMMUNE_THYROID_DISEASE
KEGG_GLYCOSPHINGOLIPID_BIOSYNTHESIS_LACTO_AND_NEOLACTO_SERIES
KEGG_DRUG_METABOLISM_CYTOCHROME_P450
KEGG_LINOLEIC_ACID_METABOLISM
KEGG_COMPLEMENT_AND_COAGULATION_CASCADES
KEGG_PENTOSE_AND_GLUCURONATE_INTERCONVERSIONS
KEGG_ASCORBATE_AND_ALDARATE_METABOLISM
KEGG_RETINOL_METABOLISM
KEGG_DORSO_VENTRAL_AXIS_FORMATION
Term
-3
0
3
6
t value of GSVA score
Group
Down
Not
Up
B
IFIT3
KEGG_CIRCADIAN_RHYTHM_MAMMAL
KEGG_VALINE_LEUCINE_AND_ISOLEUCINE_DEGRADATION
KEGG_N_GLYCAN_BIOSYNTHESIS
KEGG_TERPENOID_BACKBONE_BIOSYNTHESIS
KEGG_RNA_POLYMERASE
KEGG_VALINE_LEUCINE_AND_ISOLEUCINE_BIOSYNTHESIS
KEGG_LIMONENE_AND_PINENE_DEGRADATION
KEGG_AMINOACYL_TRNA_BIOSYNTHESIS
KEGG_NUCLEOTIDE_EXCISION_REPAIR
KEGG_MISMATCH_REPAIR
KEGG_BASE_EXCISION_REPAIR
KEGG_RIBOFLAVIN_METABOLISM
KEGG_ETHER_LIPID_METABOLISM
KEGG_STEROID_BIOSYNTHESIS
KEGG_DNA_REPLICATION
KEGG_ASTHMA
KEGG_ALPHA_LINOLENIC_ACID_METABOLISM
KEGG_PORPHYRIN_AND_CHLOROPHYLL_METABOLISM
KEGG_INTESTINAL_IMMUNE_NETWORK_FOR_IGA_PRODUCTION
KEGG_PYRIMIDINE_METABOLISM
KEGG_ALLOGRAFT_REJECTION
KEGG_TAURINE_AND_HYPOTAURINE_METABOLISM
KEGG_PRIMARY_IMMUNODEFICIENCY
KEGG_HISTIDINE_METABOLISM
KEGG_SULFUR_METABOLISM
KEGG_ANTIGEN_PROCESSING_AND_PRESENTATION
KEGG_ONE_CARBON_POOL_BY_FOLATE
KEGG_PRIMARY_BILE_ACID_BIOSYNTHESIS
KEGG_ARACHIDONIC_ACID_METABOLISM
KEGG_AUTOIMMUNE_THYROID_DISEASE
KEGG_GRAFT_VERSUS_HOST_DISEASE
KEGG_DRUG_METABOLISM_CYTOCHROME_P450
KEGG_RENIN_ANGIOTENSIN_SYSTEM
KEGG_FOLATE_BIOSYNTHESIS
KEGG_BIOSYNTHESIS_OF_UNSATURATED_FATTY_ACIDS
KEGG_MATURITY_ONSET_DIABETES_OF_THE_YOUNG
KEGG_GLYCOSAMINOGLYCAN_BIOSYNTHESIS_KERATAN_SULFATE
KEGG_HOMOLOGOUS_RECOMBINATION
KEGG_PHENYLALANINE_METABOLISM
KEGG_CELL_CYCLE
KEGG_GLYCINE_SERINE_AND_THREONINE_METABOLISM
KEGG_TYPE_I_DIABETES_MELLITUS
KEGG_SYSTEMIC_LUPUS_ERYTHEMATOSUS
KEGG_ASCORBATE_AND_ALDARATE_METABOLISM
KEGG_PENTOSE_AND_GLUCURONATE_INTERCONVERSIONS
KEGG_LINOLEIC_ACID_METABOLISM
KEGG_RETINOL_METABOLISM
KEGG_GLYCOSPHINGOLIPID_BIOSYNTHESIS_LACTO_AND_NEOLACTO_SERIES
KEGG_COMPLEMENT_AND_COAGULATION_CASCADES
KEGG_DORSO_VENTRAL_AXIS_FORMATION
Term
-5.0
-2.5
0.0
2.5
t value of GSVA score
Group
Down
Not
Up
C
IFIT1
KEGG_AMINOACYL_TRNA_BIOSYNTHESIS
KEGG_CIRCADIAN_RHYTHM_MAMMAL
KEGG_CYTOSOLIC_DNA_SENSING_PATHWAY
KEGG_REGULATION_OF_AUTOPHAGY
KEGG_RIBOSOME
KEGG_RIG_I_LIKE_RECEPTOR_SIGNALING_PATHWAY
Running Enrichment Score
0.4
0.0
-0.4
Ranked List Metric
0.50
0.25
0.00
-0.25
5000
10000
15000
20000
Rank in Ordered Dataset
D
IFIT3
KEGG_CHEMOKINE_SIGNALING_PATHWAY
KEGG_CYTOKINE_CYTOKINE_RECEPTOR_INTERACTION
KEGG_NOD_LIKE_RECEPTOR_SIGNALING_PATHWAY
KEGG_RIBOSOME
KEGG_RIG_I_LIKE_RECEPTOR_SIGNALING_PATHWAY
KEGG_TOLL_LIKE_RECEPTOR_SIGNALING_PATHWAY
Running Enrichment Score
0.6
0.3
0.0
-0.3
Ranked List Metric
0.50
0.25
0.00
-0.25
5000
10000
15000
20000
Rank in Ordered Dataset
