# Supplementary material for: Role of IFIT1 and IFIT3 in systemic lupus erythematosus: modeling a diagnosis and exploring immune regulation
Source: Clin Exp Med. 2026 Jul 24;26(1):313. doi: 10.1007/s10238-026-02265-6 (PMC13534192; doi:10.1007/s10238-026-02265-6)
Supplement: Supplementary file 5 [file 10238_2026_2265_MOESM1_ESM.docx]

| Dataset | Platform | Total | SLE | Normal |
| --- | --- | --- | --- | --- |
| GSE61635 | GPL570 | 129 | 99 | 30 |
| GSE50772 | GPL570 | 81 | 61 | 20 |

Supplementary Table 1,Systemic lupus erythematus (SLE)-related microarrays datasets
